# Stereotactic radiotherapy for patients with bone metastases: a selected group with low rate of radiation treatment during the last month of life?

**DOI:** 10.1186/s13014-024-02547-x

**Published:** 2024-11-01

**Authors:** Carsten Nieder, Ellinor C. Haukland, Luka Stanisavljevic, Bård Mannsåker

**Affiliations:** 1 Institute of Clinical Medicine, Faculty of Health Sciences, UiT – The Arctic University of Norway, Tromsø, Norway; 2 Department of Oncology and Palliative Medicine, Nordland Hospital, 8092 Bodø, Norway; 3 Department of Quality and Health Technology, SHARE – Center for Resilience in Healthcare, Faculty of Health Sciences, University of Stavanger, Stavanger, Norway

**Keywords:** Palliative radiation therapy, Stereotactic radiotherapy, Overtreatment, Quality of care, Treatment completion, End of life care, Fractionation, Metastatic cancer

## Abstract

**Background:**

Complex high-precision radiotherapy, such as stereotactic body radiotherapy (SBRT), should only be offered to patients with sufficiently long survival. In the context of bone metastases radiotherapy, low rates of treatment close to the end of life, e.g. last 30 days (RT30), may serve as a quality of care indicator. While traditional, pain-relieving short-course regimens have been studied comprehensively, real-world SBRT results are still limited.

**Methods:**

Retrospective analysis (2010–2023, n = 1117 episodes) of patients with bone metastases treated with traditional single-fraction (8 Gy × 1) or multi-fraction regimens (often 4 Gy × 5 or 3 Gy × 10) compared to stereotactic single-fraction (12–16 Gy × 1) or multi-fraction regimens.

**Results:**

Except for gender, almost all baseline variables were uneven distributed. Failure to complete fractionated radiotherapy was uncommon in the stereotactic (4%) and non-stereotactic group (3%), *p* = 1.0. With regard to RT30, relevant differences emerged (19% for 8-Gy single-fraction versus 0% for stereotactic single-fraction, *p* = 0.01). The corresponding figures were 11% for multi-fraction non-stereotactic and 2% for multi-fraction stereotactic, *p* = 0.08. Median overall survival was shortest after 8-Gy single-fraction irradiation (4.2 months) and longest after stereotactic multi-fraction treatment (13.9 months). Neither stereotactic radiotherapy nor multi-fraction treatment improved survival in multivariate Cox regression analysis. Factors significantly associated with longer survival included better performance status, lower LabBM score (5 standard blood test results), stable disease outside of irradiated area(s), metachronous distant metastases, longer time interval from metastatic disease to bone irradiation, and outpatient status.

**Conclusion:**

The implementation of SBRT for selected patients has resulted in low rates of non-completion and RT30. Optimal selection criteria remain to be determined, but in current clinical practice we exclude patients with poor performance status, unfavorable blood test results (high LabBM score) and progressive disease sites not amenable to SBRT. Established, guideline-endorsed short-course regimens, especially 8-Gy single-fraction treatment, continue to represent an important palliative approach.

## Introduction

Stereotactic radiotherapy has demonstrated its potential in several indications and treatment sites, e.g., brain and lung [1]. High precision, limited toxicity and effective local control are hallmarks of this type of external beam radiotherapy [2]. Painful uncomplicated bone metastases have long been treated with simple short-course regimens, which continue to represent the cornerstone today, e.g., a single fraction of 8 Gy [3]. Other bone metastases scenarios often require different, sometimes multi-modal approaches. Complicated or post-surgical bone metastases may preferably receive higher doses of radiation, historically consisting of 10–13 fractions [4]. In this context, stereotactic radiotherapy has the potential to shorten treatment time [5–9]. A third scenario consists of oligometastatic spread, where ablative doses of radiation may lead to durable local control and often long survival [1, 10]. Typically, hypofractionation or single doses are also administered to these patients. As a result of widespread availability of equipment and expertise, including recent practice recommendations [11, 12], radiotherapy of bone metastases has become a more individualized approach.

Nguyen et al. performed a randomized phase 2 trial of single-fraction stereotactic versus conventional multi-fraction radiotherapy for pain relief in patients with predominantly non-spine bone metastases [9]. Patients were randomly assigned in a 1:1 ratio to receive either single-fraction treatment (12 Gy for ≥ 4-cm lesions or 16 Gy for < 4-cm lesions) or 30 Gy in 10 fractions. Among evaluable patients who received treatment per protocol, the single-fraction group had more pain responders than the control group (complete + partial response at 3 months 72% versus 49%, *p* = 0.03). No differences were found in treatment-related toxic effects or quality-of-life scores. Local control rates at 1 and 2 years were higher in patients receiving single-fraction radiotherapy. These promising results led our group to adopt this treatment approach, complementing already existing fractionated regimens. Given that 16 Gy still results in a moderate biologically equivalent dose in 2-Gy fractions (EQD2) to tumor cells with high α/β value [13], we preferred hypofractionated regimens such as 12 Gy × 2 or 9 Gy × 3 (higher EQD2) in selected patients with presumed benefit and longer survival. In parallel, standard palliative conventional techniques remained in clinical use in the majority of patients [14].

In the same time period, our group has performed extensive studies of prognostic factors for survival and factors predicting futile radiotherapy, defined as radiotherapy very close to the end of life, e.g. last 30 days (RT30) [15–17]. We became increasingly confident in blood biomarker-based predictive and prognostic models such as the Glasgow prognostic score (C-reactive protein (CRP) and albumin) [18] and the LabBM score (CRP, albumin, hemoglobin, platelets, lactate dehydrogenase (LDH)) [19] and expanded the latter by including performance status (PS), resulting in the LabPS score [16]. We did not formally require a certain prognostic score, e.g., favorable LabBM or LabPS, when offering stereotactic radiotherapy, as additional factors often contribute important information, e.g., availability of and eligibility for systemic therapy and multidisciplinary input [20]. However, we hypothesized that our clinical focus on assessment of various factors, including but not limited to blood tests, may have resulted in lower than typically reported rates of radiotherapy in the last month of life in patients treated with more complex and resource consuming stereotactic approaches. Therefore, we performed the present retrospective single-institution study.

## Patients and methods

A retrospective study was performed at Nordland Hospital Bodø employing the following inclusion criteria: adult patients, irradiation of bone metastases in routine clinical practice outside of prospective trials, and consecutively treated in the time period January 01, 2010–May 31, 2023. Patients with hematological primary diagnosis were excluded, e.g., multiple myeloma. A total of 1117 treatment episodes were analyzed, meaning that some patients returned for irradiation of previously unirradiated bone metastases and others for in-field re-irradiation. The hospital’s clinical oncologists prescribed both radiotherapy and systemic treatment, guided by tumor-specific national guidelines and multidisciplinary tumor board discussions. Decision-making was individualized without employing a certain prognostic score as a stop or go criterion. However, PS and blood test results were utilized at physician’s discretion to inform choice of radiotherapy fractionation and technique.

Stereotactic treatment started in 2017, initially hypofractionated, later also as a single-fraction regimen. The highest share of stereotactic treatment was observed in 2020 (26%), likely due to COVID-19-related policy changes. However, the proportion remained relatively stable, e.g., 22% in 2022. Intensity-modulated or volumetric modulated arc techniques were employed, while simpler 3-D techniques remained in use for non-stereotactic treatment. Single-fraction stereotactic treatment (n = 27) was guided by the study reported by Nguyen et al. [9]. Fractionated stereotactic treatment (n = 48) was preferred in patients with better prognosis and individualized as needed to adhere to safe dose constraints. Fifty-eight percent of courses employed > 5 fractions and 42% 2–5 fractions. Non-stereotactic treatment (n = 1042) consisted of short-course (8 Gy × 1 or 4 Gy × 5) in 43%, 3 Gy × 10 (49%) or protracted course, e.g., 3 Gy × 13, in 8%.

Descriptive statistics and 2-tailed Fisher’s exact probability tests were employed for statistical analyses in IBM SPSS statistics 28 (IBM SPSS Statistics, Somers, NY, USA). Survival data were obtained in October 2023 by use of the hospital’s electronic patient records. The latter were also utilized to extract baseline characteristics. The number of censored observations (alive in October 2023) was 126 (11%). Date of death was known in all remaining patients. Actuarial survival curves were calculated according to the Kaplan–Meier method and analyzed by log-rank tests (start date: first fraction of radiotherapy). Furthermore, forward conditional Cox regression analyses were employed. Statistical significance was defined as *p*-value < 0.05.

Finally, to account for systematic differences in baseline characteristics between the stereotactic and non-stereotactic treatment groups, a propensity score matched analysis was employed. The propensity score was defined as the probability that an individual would have been allocated to the stereotactic treatment group as a function of observed baseline characteristics, estimated using multivariable logistic regression in which the treatment group was the dependent variable and the baseline characteristics were the independent variables. Due to the large number of available non-stereotactic patients, we removed those treated before 2017 and those with Karnofsky PS (KPS) < 50 from the database (all stereotactic patients had higher KPS). Although 1:1 matching is the most common method, our study was large enough to use more than one control patient in many-to-one matching (often 2–4). Nearest neighbor matching was employed, where the difference in propensity scores that range from 0–1 was minimized within each matched sample based on a threshold of 0.1.

## Results

The results were stratified by treatment regimen (single fraction or not, stereotactic or not), resulting in 4 strata (Table 1). Except for gender, almost all baseline variables were uneven distributed. The stereotactic groups contained fewer patients with breast and more patients with kidney cancer. The stereotactic single-fraction group had a high proportion of patients with femoral metastases (37%). Stereotactically treated patients were less likely to receive opioid analgesics and more likely to receive outpatient radiotherapy. They were also less likely to harbor progressive disease sites that were not included in the actual course of radiotherapy. KPS was different too (best in patients with fractionated stereotactic radiotherapy, at least 50 in all patients treated stereotactically, minimum 30 in other patients). Comparable differences were seen for the LabBM score (CRP, albumin, hemoglobin, platelets, LDH) [19], i.e. best prognosis in the stereotactic fractionated group and no stereotactically treated patients with a score of 3.5 (highest possible point sum, ranging from 0 to 3.5, with 3.5 meaning that all 5 blood tests were abnormal).

**Table 1:** Comparison between treatment episodes where patients were treated with traditional palliative radiotherapy (8 Gy single fraction or multiple fractions) or with stereotactic radiotherapy regimens (12-16 Gy single fraction or multiple fractions): baseline parameters

Parameter	8 Gy single fraction (number, percent)	Multiple fractions, non-stereotactic	12–16 Gy single fraction	Multiple fractions, stereotactic	*p*-value, all 4 strata
*Gender*					
Female sex	84, 36	281, 35	11, 41	11, 23	0.31
Male sex	149, 64	528, 65	16, 59	37, 77	
*Primary disease site*					
Breast cancer	44, 19	142, 18	3, 11	2, 4	< 0.001
Prostate cancer	95, 41	251, 31	6, 22	17, 35	
SCLC	13, 6	20, 3	0, 0	2, 4	
NSCLC	28, 12	165, 20	9, 33	9, 19	
Kidney cancer	9, 4	60, 7	3, 11	11, 23	
Bladder cancer	2, 1	22, 3	2, 4	0, 0	
Colorectal cancer	18, 8	59, 7	3, 11	3, 6	
Malignant melanoma	4, 2	16, 2	0, 0	1, 2	
Others	20, 9	74, 9	1, 4	3, 6	
*Site of treated metastasis*^1^					
Spine	122, 52	522, 65	9, 33	21, 44	< 0.001
Pelvis	119, 51	367, 45	12, 44	15, 31	0.08
Shoulder	43, 19	61, 8	1, 4	4, 8	< 0.001
Rib	33, 14	94, 12	8, 30	13, 27	< 0.001
Femur	28, 12	89, 11	10, 37	2, 4	< 0.001
Other parameters					
Inpatient	63, 27	271, 34	4, 15	2, 4	< 0.001
Not on opioid analgesics	55, 24	324, 40	14, 52	32, 67	< 0.001
Not on systemic therapy	41, 18	229, 28	8, 30	15, 31	0.009
Progressive disease^2^	128, 55	352, 44	11, 41	7, 15	< 0.001
Re-irradiation	46, 20	120, 15	6, 22	2, 4	0.03
Early irradiation (2 mo)^3^	33, 14	262, 32	8, 30	15, 31	< 0.001
Synchronous metastases^4^	110, 47	408, 51	17, 63	16, 34	0.06
Median time from metastatic cancer (months), range	14, 1–170	8, 1–180	10, 1–168	6, 1–108	0.03
Median age (years), range	73, 26–95	69, 34–92	73, 51–90	72, 24–86	< 0.001
Median Karnofsky performance status, range	70, 30–100	70, 30–100	70, 50–100	90, 60–100	< 0.001
Median LabBM score, range	2.0, 0–3.5	1.5, 0–3.5	1.5, 0–3.0	1.0, 0–3.0	< 0.001

SCLC: small cell lung cancer, NSCLC: non-small cell lung cancer

^1^not displayed: sternum, humerus, skull and other uncommon targets

^2^outside of irradiated region(s)

^3^in the first 2 months after cancer diagnosis

^4^present already when diagnosed with cancer

Failure to complete fractionated radiotherapy was uncommon in the stereotactic (4%) and non-stereotactic group (3%), *p* = 1.0. With regard to RT30, relevant differences emerged (19% for 8-Gy single-fraction versus 0% for stereotactic single-fraction, *p* = 0.01). The corresponding figures were 11% for fractionated non-stereotactic and 2% for fractionated stereotactic, *p* = 0.08 (Table 2). Two percent in the fractionated stereotactic group actually represents a single patient. He was 74 years old (outpatient, KPS 70, LabBM 2.0) and had kidney cancer with metachronous metastases (12 months from diagnosis of metastatic disease, not on systemic therapy). He was hospitalized for bowel perforation from diverticulitis, i.e. not treatment-related, and died in the early post-operative phase from COVID-19. It is of course impossible to predict this type of acute health deterioration with fatal outcome. In our study, rates of RT30 remained stable over time.

**Table 2:** Comparison between treatment episodes where patients were treated with traditional palliative radiotherapy (8 Gy single fraction or multiple fractions) or with stereotactic radiotherapy regimens (12–16 Gy single fraction or multiple fractions): outcomes

Parameter	8 Gy single fraction (number, percent)	Multiple fractions, Non-stereotactic	12–16 Gy single fraction	Multiple fractions, stereotactic	p-value
PRT30	44, 19	87, 11	0, 0	1, 2	0.01 (single fr.)
					0.08 (multiple fr.)
Incomplete PRT	0, 0	28, 3	0, 0	2, 4	1.0*
Alive 1 year after PRT (%)	23	39	37	58	Calculated for complete curves, see Fig. 1

PRT30: palliative radiotherapy in the last month of life

^*^for the 2 groups with multiple fractions (incomplete single fraction irradiation is not possible)

**Fig. 1:**
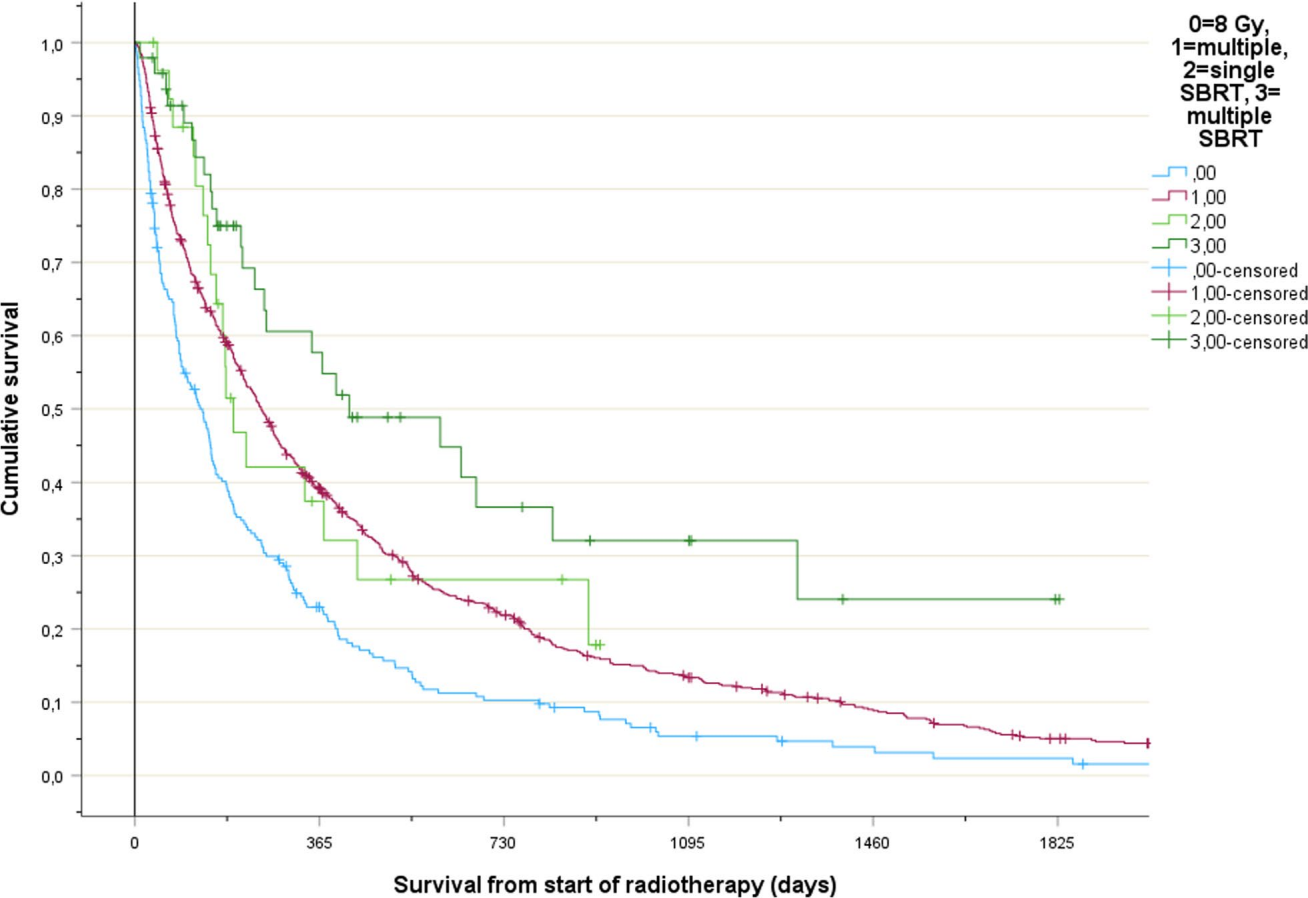
Actuarial overall survival (Kaplan–Meier curves), *p* < 0.001 (pooled over all strata). 8-Gy single-fraction was associated with significantly shorter survival than all 3 other regimens (*p* = 0.025, < 0.001, and < 0.001, respectively). Fractionated stereotactic radiotherapy was associated with longer survival than fractionated non-stereotactic, *p* = 0.004. The difference between the 2 stereotactic groups was not significant, *p* = 0.13

Median overall survival was shortest after 8 Gy single-fraction irradiation (4.2 months, 95% confidence interval (CI) 3.1–5.3). Stereotactic single-fraction irradiation resulted in 6.4 months (95% CI 4.4–8.4). The corresponding figures were 8.2 (7.3–9.1) and 13.9 (3.5–24.4) for fractionated non-stereotactic and stereotactic, respectively (Fig. 1). Univariate Cox regression analyses for continuous variables showed significant associations with improved overall survival for better KPS (*p* < 0.001), lower LabBM score (*p* < 0.001), and longer time from metastatic disease to radiotherapy for bone metastases (*p* = 0.02), but not age and year of radiotherapy. Dichotomized variables were evaluated by log-rank tests, which showed that inpatient status (*p* < 0.001), progressive disease outside of irradiated area(s) (*p* < 0.001), and synchronous distant metastases at first cancer diagnosis (*p* < 0.001) were associated with shorter overall survival. All these parameters were then included in a multivariate model. Neither stereotactic radiotherapy nor multi-fraction treatment improved survival in multivariate Cox regression analysis. Factors significantly associated with longer survival included better KPS (*p* < 0.001; continuous variable), lower LabBM score (*p* < 0.001; continuous variable), stable disease outside of irradiated area(s) (*p* < 0.001; yes/no), metachronous distant metastases (*p* = 0.002; yes/no), longer time interval from metastatic disease to bone irradiation (*p* = 0.007; continuous variable), and outpatient status (*p* = 0.025; yes/no).

With regard to propensity score matched analyses, we evaluated 305 patients that were matched according to outpatient status, LabBM score, stable disease outside of irradiated area(s), KPS and tumor type. As shown in Table 3, similar rates of RT30 were observed (0–5%). One-year survival rates were similar too, both within the single fraction (37 and 41%, respectively) and the fractionated subset (58%, identical).

**Table 3:** Comparison between treatment episodes where patients were treated with traditional palliative radiotherapy (8 Gy single fraction or multiple fractions) or with stereotactic radiotherapy regimens (12–16 Gy single fraction or multiple fractions): outcomes of matched analyses in 305 patients with comparable prognostic features

Parameter	8 Gy single fraction (number, percent)	Multiple fractions, Non-stereotactic	12–16 Gy single fraction	Multiple fractions, stereotactic	p-value
PRT30	1, 2	9, 5	0, 0	1, 2	0.3–0.45
Incomplete PRT	0, 0	4, 2	0, 0	2, 4	1.0*
Alive 1 year after PRT (%)	41	58	37	58	Calculated for complete curves

PRT30: palliative radiotherapy in the last month of life

^*^for the 2 groups with multiple fractions (incomplete single fraction irradiation is not possible)

## Discussion

This retrospective single-institution study analyzed our experience with more complex and resource consuming stereotactic approaches for radiotherapy of bone metastases. We were interested in patient selection, treatment completion, radiotherapy very close to the end of life (RT30), and overall survival. Other endpoints such as pain relief, local control, fracture rates, toxicity and quality of life were not evaluated. However, recent reviews and meta-analyses of prospective clinical studies have already provided information about these endpoints [2, 5, 6, 10]. When introducing stereotactic radiotherapy for bone metastases in 2017, an active research program was already running, focusing on improved patient selection for palliative radiotherapy and development of better decision support tools. More recently, these efforts led to recommendation of the LabBM score [15], followed by the LabPS score [16]. In addition, a model named palliative appropriateness criteria score (PACS) contributes by estimating the proportion of remaining lifetime spent on radiotherapy [20, 21]. During this work-in-progress phase, we did not feel sufficiently confident to implement one of the scores as obligatory requirement for decision making. We did, however, try to select patients with poor prognostic features for short-course non-stereotactic treatment, especially 8 Gy × 1, by assessing KPS, disease extent, remaining systemic therapy options and blood test results.

The comparison of baseline parameters (Table 1) confirmed that major differences in prognostic factors were present. Obviously, patients were assigned to single-fraction treatment if adverse factors were found and to multiple fractions if expected survival was longer, with some individual variation and sometimes imperfect match between survival and time on radiotherapy. Fewer stereotactically treated patients received opioid analgesics and inpatient radiotherapy. They were also less likely to harbor progressive disease sites that were not included in the actual course of radiotherapy. KPS was different too (best in patients with fractionated stereotactic radiotherapy). Comparable differences were seen for the LabBM score, i.e. best prognosis in the stereotactic fractionated group. Better patient selection resulted in lower rates of RT30. There is little, if any, potential for further improvement of RT30 rates in our stereotactic radiotherapy groups. However, adhering to thorough prognostic assessment is expected to improve quality of care in the non-stereotactic groups by lowering RT30 rates in the future. The policy in place during the time period of the study resulted in stable rates of RT30 over time. Given the group’s research focus on prognostic models, this lack of positive development is surprising. On the other hand, offering 8 Gy × 1 is neither very resource-consuming nor toxic, and is more polite and supportive than refusing radiotherapy at all. This does not automatically justify the decision, however, many clinicians may prefer treatment, especially if they have limited training in palliative care and the patient’s appointment is radiotherapy-specific.

Gillespie et al. studied almost 3000 patients who received radiotherapy for bone metastases at a different institution in the 3-year time period between 2016 and 2018 [7]. Their analysis included close to 6000 radiotherapy episodes. SBRT was frequently employed (n = 2790, 47%), while 8 Gy × 1 was infrequent (n = 368, 6%). On multivariate logistic regression, factors associated with receipt of SBRT were high KPS, a non-radiosensitive primary tumor histology, and location in spine. Death within 30 days occurred among 24% of all 8 Gy × 1 treatments (19% in our study), 9% of 3 Gy × 10 courses, and 4% of all SBRT treatments (*p* < 0.01). Outside of our center, the LabBM score has so far not been studied specifically in the bone metastases setting. Our initial experience in this large cohort supports further validation by other groups.

Kowalchuk et al. developed and validated a decision-making tool predictive of overall survival for patients receiving SBRT for spinal metastases (single or multiple fractions), i.e. a subgroup of all bone SBRT [22]. Three hundred sixty-one patients at one institution were used for the training set, and 182 at a second institution were used for external validation. The final model consisted of the following variables and scores: Spinal Instability Neoplastic Score (SINS) ≥ 6 (1), time from primary diagnosis < 21 months (1), Eastern Cooperative Oncology Group (ECOG) PS = 1 (1) or ECOG PS > 1 (2), and > 1 organ system involved (1). Each variable was an independent predictor of survival (*p* < 0.001), and each 1-point increase in the score was associated with a hazard ratio of 2.0. Three groups were defined: favorable (0–1 points), intermediate (2 points), and poor survival (3–5 points), with 2-year survival rates of 84, 46 and 21%, respectively (*p* < 0.0001 for each). In the external validation set, the score was also predictive of survival. Just like Gillespie et al. [7], Kowalchuk et al. did not include blood test results. The same is true for Zeng et al. [23], who also focused on spine SBRT and prognostic factors associated with surviving less than 3 months versus greater than 3 years, meaning that different endpoints were selected in all studies.

At our center, median overall survival was 6.4 months after stereotactic single-fraction irradiation. Largely identical results were reported by Nguyen et al. (median 6.7 months in both arms of their randomized study (comparator: conventional multi-fraction irradiation) [9]. In our study, the corresponding figure was 8.2 months for conventional multi-fraction treatment. Multivariately, neither fractionation nor stereotactic treatment was associated with improved survival. Propensity score matching also confirmed similar survival outcomes. Therefore, if the primary aim of treatment is symptom palliation, priority is given to simple short-course regimens, which avoid toxicity and may be applied in a fast-track setting. Single-fraction or 2–3 fraction stereotactic radiotherapy is a promising alternative, but less easy to plan and deliver. In addition, in a larger randomized clinical trial than the one reported by Nguyen et al. superiority of stereotactic single-fraction irradiation for the primary end point of patient-reported pain response at 3 months was not found [24]. These researchers studied single-fraction doses of 16 or 18 Gy versus a conventional 8-Gy regimen, limited to spine metastases.

The present study has both strengths, such as the routine clinical practice setting and availability of blood test results, and limitations, such as the moderate number of stereotactic radiotherapy episodes, lack of other outcome data, and limited follow-up if treatment was administered after 2021. There is still debate about complete avoidance of palliative radiotherapy in the last month of life. Recently, Christ et al. reported that radiotherapy achieved high completion and success rates until one week before death, and suggested that treatment within one week of death should be restricted to carefully selected patients or avoided altogether [25]. In a study by Rautakorpi et al., treatment was discontinued in 41% of the patients irradiated during the last two weeks of life, and worsening of the general condition was the prevailing reason for discontinuation (70%) [26]. Despite controversy, RT30 has remained the most common quality of care indicator [27–29]. In a limited number of bone metastases treatment episodes (< 300), we recently identified three significant predictors of 30-day mortality (KPS (≤ 50, 60–70, 80–100), weight loss of at least 10% within 6 months (yes/no), pleural effusion (present/absent)) and employed these to construct a predictive model with 5 strata and mortality rates of 0–75% [17]. Future validation in a larger study with additional potential predictors is planned.

## Conclusion

The implementation of stereotactic radiotherapy for selected patients has resulted in low rates of non-completion and RT30. Optimal selection criteria remain to be determined, but in current clinical practice we exclude patients with poor performance status, unfavorable blood test results (high LabBM score) and progressive disease sites not amenable to SBRT. Established, guideline-endorsed short-course regimens, especially 8-Gy single-fraction treatment, continue to represent an important palliative approach.

## Data Availability

No datasets were generated or analysed during the current study.

## Abbreviations

IMRT: Intensity-modulated radiotherapy
VMAT: Volumetric modulated arc treatment
SBRT: Stereotactic body radiotherapy
EQD2: Equivalent dose in 2-Gy fractions
RT30: Palliative radiotherapy in the last 30 days of life
LabBM: Laboratory values in brain metastases
